# Hepatocyte-derived Pumilio1-enriched exosomes inhibit HSC activation by suppressing tropomyosin-4 translation

**DOI:** 10.1097/HC9.0000000000000759

**Published:** 2025-07-14

**Authors:** Zhiping Wan, Xiang Cai, Xiaoquan Liu, Haoqi Chen, Xiaoman Chen, Xiaoan Yang, Qingqing Feng, Hong Cao, Qiyi Zhao, Hong Deng

**Affiliations:** 1 Department of Infectious Diseases, The Third Affiliated Hospital of Sun Yat-sen University, Guangzhou, China; 2 Guangdong Key Laboratory of Liver Disease Research, The Third Affiliated Hospital of Sun Yat-sen University, Guangzhou, China; 3 Department of Hepatic Surgery, Liver Transplantation, The Third Affiliated Hospital of Sun Yat-sen University, Guangzhou, China

**Keywords:** crosstalk, extracellular vesicles, fatty liver, liver injury, RNA binding proteins

## Abstract

**Background:**

Metabolic dysfunction–associated steatotic liver disease (MASLD) has become the most common chronic liver disease globally. Abnormal crosstalk between hepatocytes and HSCs leads to liver fibrosis and aggravates MASLD. We explored the role of the RNA-binding protein Pumilio in this process.

**Methods:**

Two isoforms of Pumilio proteins (PUM1, PUM2) expression were analyzed in the livers of MASLD patients and mice. MASLD mice were induced by a western diet combined with intraperitoneal injection of carbon tetrachloride (WD+CCl_4_), or a choline-deficient, L-amino acid–defined, high-fat diet (CDAHFD). Adeno-associated virus type 8 carrying Pum1-targeting short hairpin RNA or small interfering RNA targeting PUM1 was used to knock down PUM1 in vivo or in vitro. Ultracentrifugation was used to isolate exosomes from cells and serum. RNA sequencing and RNA immunoprecipitation experiments were used to find and identify the target genes of PUM1.

**Results:**

The expression of PUM1, not PUM2, was decreased in both MASLD patients and models. PUM1 knockdown aggravated liver injury. PUM1 also decreased in steatotic hepatocytes. Upregulating PUM1 improved lipid deposition and reduced hepatocyte lipotoxic death. Hepatocytes regulate the activation of HSCs by PUM1-enriched exosomes. Tropomyosin 4 (TPM4) was identified as a target of PUM1. PUM1 affected the expression of TPM4 by binding to its mRNA, thereby regulating HSCs activation.

**Conclusions:**

While PUM1 is downregulated during MASLD progression, upregulation of PUM1 improves lipid deposition, reduces hepatocyte lipotoxic death and inhibits TPM4 expression to reduce HSC activation.

## INTRODUCTION

Globally, over a third of adults suffer from metabolic dysfunction–associated steatotic liver disease (MASLD).^1^ This disorder is characterized by hepatic steatosis similar to NAFLD, but with additional cardiometabolic risk factors.^2^ Excessive lipid accumulation causes chronic liver injury and inflammatory responses that then trigger the activation of HSCs.^3^ In addition, exosomes play a role in the activation of HSCs.^4^ These activated HSCs synthesize and secrete large amounts of collagen-rich extracellular matrix, a major contributor to liver fibrosis during MASLD progression.^5^ Liver fibrosis itself is a dangerous condition that accelerates the development of cirrhosis and HCC.^3^^,^^5^ The severity of liver fibrosis is positively associated with all-cause and liver-related mortality in patients with MASLD.^6^ However, treatment options are limited because of an insufficient understanding of the mechanisms behind MASLD-related liver fibrosis.

RNA-binding proteins have specific domains targeting various RNAs that regulate metabolic processes.^7^ Regulation occurs through mediating RNA translation, maturation, transport, and localization.^7^ One highly conserved RNA-binding protein is Pumilio (PUM), part of the pumilio–fem3 binding factor family. PUM1 and PUM2 are its 2 isoforms.^8^ Both are important to tissue and organ development, immune regulation, and tumor progression.^9^^–^^11^ PUM1 or PUM2 affects the cell cycle and proliferation by inhibiting the translation of cyclin-dependent kinase inhibitor 1B (CDKN1B).^12^ PUM1 also regulates hemoglobin and exerts anti-aging and anti-inflammatory effects via binding to mRNA encoding toll-like receptor 4 (TLR4).^13^^,^^14^ In contrast, PUM2 inhibits the expression of mitochondrial fission factors, impairing mitochondrial fission and mitophagy.^15^ PUM2 downregulation promotes bone regeneration and prevents osteoporosis.^16^ PUM1 is involved in regulating metabolic reprogramming, and the deficiency of PUM1 inhibits glycolytic metabolism.^17^ PUM1 also regulates the PERK/eIF2/ATF4 signaling pathway,^18^ which is closely associated with the progression of MASLD.^19^ While both PUM isoforms may play a role in MASLD liver injury, no study to date has investigated that role or any regulatory targets of the 2 proteins. The lack of understanding of MASLD pathogenesis limits the development of effective treatments.

To address this gap in knowledge, here, we aimed to determine PUM1/2 expression levels in MASLD using a combination of public databases, patient liver samples, and animal models. To verify the function of these proteins and understand their mechanisms, we also performed knockdown experiments in vivo and in vitro, and functional analyses on genes analyzed by RNA sequencing. Our findings contribute to existing research on the pathogenesis of MASLD and provide potential biological targets for novel treatments.

## METHODS

### Human datasets and liver tissue

*PUM1* and *PUM2* mRNA expression in liver of MASLD patients, as well as in activated HSCs, was analyzed through using the following Gene Expression Omnibus datasets: GSE126848,^20^ GSE24807,^21^^,^^22^ GSE17470,^23^ and GSE128940.^24^ Human liver tissue was collected from liver biopsy or surgical specimens at the Third Affiliated Hospital of Sun Yat-Sen University, with informed consent from the patients and the procedures were approved by the Medical Ethics Committee of said hospital (approval number: [2018]02-337-01). All research was conducted in accordance with the Declarations of Helsinki.

### Animal experiments

Male C57BL/6 (6–8-week-old) mice were obtained from Guangzhou University of Chinese Medicine (Guangzhou, China) and housed in a barrier environment under a 12-h light/12-h dark cycle. Food and water access were ad libitum.

After 1 week of adaptive feeding, 8-week-old mice were randomly and blindly allocated to either the control or MASLD groups. Mice in the MASLD group were subjected to a western diet (WD) (#TD.120528, Teklad Custom Diet) with high-sugar water (18.9 g/L d-glucose and 23.1 g/L d-fructose) and weekly intraperitoneal (i.p.) injections of carbon tetrachloride (CCl_4_, 0.2 μL/g of body weight) till they were 20 weeks old. Over the same period, control mice were fed a normal diet (#MD17121, Mediscience) with untreated water and subjected to weekly i.p. injections of corn oil.

Similarly, after 1 week of adaptive feeding, 6-week-old male C57BL/6 mice were split into control or MASLD groups. Mice in the MASLD group were subjected to a choline-deficient, L-amino acid–defined, high-fat diet (CDAHFD) (#A06071302, Research Diets) till they were 16 weeks old. Control mice were fed a normal diet (#MD17121, Mediscience) over the same period.

An adeno-associated virus type 8 system carrying shRNA targeting *Pum1* (AAV8-shPum1, Genechem) or a negative control (AAV8-shNC, Genechem) was used to knock down PUM1 expression in the mouse liver. When WD+CCl_4_ mice reached 15 weeks of age, they were injected with 2.5 × 10^11^ AAV8-shPum1 or AAV8-shNC through the tail vein. The CDAHFD mice received the same injections at 11 weeks of age. Both groups then progressed with the MASLD induction treatment till the mice were 20 weeks old for the former and 16 weeks old for the latter.

Next, mice were fasted overnight before euthanasia for collection of blood, liver, heart, lungs, kidneys, brain, and testes. All animal experiments were approved by the Animal Ethics Committee (approval numbers: IACUC-AEWC-F2305010 and IACUC-AEWC-F2308003).

### Histopathological analysis

Liver tissues were embedded in paraffin, deparaffinized with xylene, and rehydrated using a graded ethanol series. Deparaffinized liver sections were stained with hematoxylin (#G1004; Servicebio) and eosin (#G1003, Servicebio) as well as Sirius Red (#GP1033; Servicebio). Fresh liver tissue was flash-frozen in liquid nitrogen, then sectioned for Oil Red O staining (#G1016, Servicebio) and hematoxylin counterstaining. Images were captured under a fluorescence microscope (Leica) and were analyzed by ImageJ software (NIH).

### Cell culture and treatment

HepG2 cells, LX-2 cells, and JS-1 cells were incubated (5% CO2, 37 °C) in high-glucose Dulbecco modified Eagle medium (#C11995500BT, Gibco) containing 10% fetal bovine serum (#10270-106, Gibco). THP-1 cells were incubated (5% CO2, 37 °C) in RPMI-1640 medium (#C11875500BT, Gibco) containing 10% fetal bovine serum (#10270-106, Gibco). The isolation and culture of primary mouse hepatocytes and primary HSCs were performed as previously described.^25^^,^^26^ In brief, the liver was digested into a single-cell suspension using collagenase. Primary mouse hepatocytes were collected by centrifuging the single-cell suspension. Primary HSCs were collected after density gradient centrifugation of the supernatant.

HepG2 cells were induced to steatotic hepatocytes by 0.3 mM palmitic acid (PA) for 24 hours. Primary mouse hepatocytes were treated with 0.2 mM PA overnight to induce steatotic hepatocytes. THP-1 cells were treated with 100 ng/mL PMA (#P8139, Sigma-Aldrich) for 24 hours to induce differentiation into macrophages. The macrophages or LX-2 cells were treated with 0.3 mM PA for 24 hours. To activate LX-2 cells, they were treated with 10 ng/mL TGF-β1 (#100-21-10, Peprotech) for 48 hours.

For knockdown experiments in HepG2 cells or LX-2 cells, 150 nM small interfering RNA (siRNA) targeting PUM1 (si-PUM1) (#siBDM2500, Ribo Biotechnology) or TPM4 (si-TPM4) (#siB10005, Ribo Biotechnology) was transfected into cells using a transfection reagent (#C10511-1, Ribo Biotechnology). For overexpression experiments, 300 ng/mL PUM1-overexpressing plasmid or 200 ng/mL TPM4-overexpressing plasmid was transfected into cells using another transfection reagent (#3000015; Thermo Fisher Scientific).

For knockdown experiments in primary mouse hepatocytes or JS-1 cells, 200 nM siRNA targeting Pum1 (#IGE2022122602, IGE Biotechnology) were transfected into cells using a transfection reagent (#3000015; Thermo Fisher Scientific). For overexpression experiments, 200 ng/mL Pum1-overexpressing plasmid was transfected into cells using transfection reagent (#3000015; Thermo Fisher Scientific).

### Cell death assays

Cell death assays were analyzed by propidium iodide (PI) staining. The cells were treated with trypsin and then incubated with PI staining solution (#HY-D0815; Med Chem Express) according to the manufacturer's instructions. Finally, the CytoFLEX LX flow cytometer was used for detection.

### Exosome isolation and identification

Exosomes were collected from the serum and medium of cells by ultracentrifugation as previously reported.^27^ The exosomes were identified by transmission electron microscopy, nanoparticle tracking analysis, and western blot analysis.

### Transcriptome sequencing

LX-2 cells were transfected (#C10511-1, Ribo Biotechnology) with 150 nM si-PUM1 (#siBDM2500, Ribo Biotechnology) or a negative control (si-NC). Total RNA was extracted and subjected to a standard Illumina transcriptome sequencing pipeline. The criteria for differentially expressed RNAs were |log2(FC)|>1 and *p*<0.05. The sequencing data were uploaded to GEO database (GSE295362).

### RNA immunoprecipitation

According to the manufacturer’s instructions (#Bes5101, BersinBio), after treatment with a polysome lysis buffer, lysed LX-2 cells were mixed with DNase salt stock, DNase, EDTA, EGTA, and DTT to remove DNA. Cell lysates were then immunoprecipitated overnight with anti-PUM1 antibody (1:20, #49885, Signalway Antibody) or IgG at 4 °C. The immunoprecipitate was incubated for 1 hour with protein A/G magnetic beads at 4 °C and then extracted using a magnetic stand. The samples were then divided for western blot analysis and RNA extraction. Western blot experiments were used to confirm the immunoprecipitation of the target protein PUM1. RNA was extracted and analyzed on the 3 groups of samples (Input, IgG, and immunoprecipitation), with further assessment carried out through quantitative reverse transcription polymerase chain reaction (RT-qPCR) experiments.

### Other experimental details

Other experimental details, such as immunofluorescence staining, immunohistochemistry staining, ALT and AST measurement, cell BODIPY staining analysis, western blot analysis, and RT-qPCR analysis, were described in the Supplemental Materials and Methods, http://links.lww.com/HC9/C38​​​​​​.

### Statistical analyses

Data are expressed as means ± SEM and analyzed in GraphPad Prism 8.0 (GraphPad Software Inc.). Between-group differences were determined using Student *t* test or 1-way ANOVA. Relationships between variables were assessed using the Spearman rank correlation test. Statistical significance was set at *p*<0.05.

## RESULTS

### PUM1 was downregulated in MASLD

**FIGURE 1:**
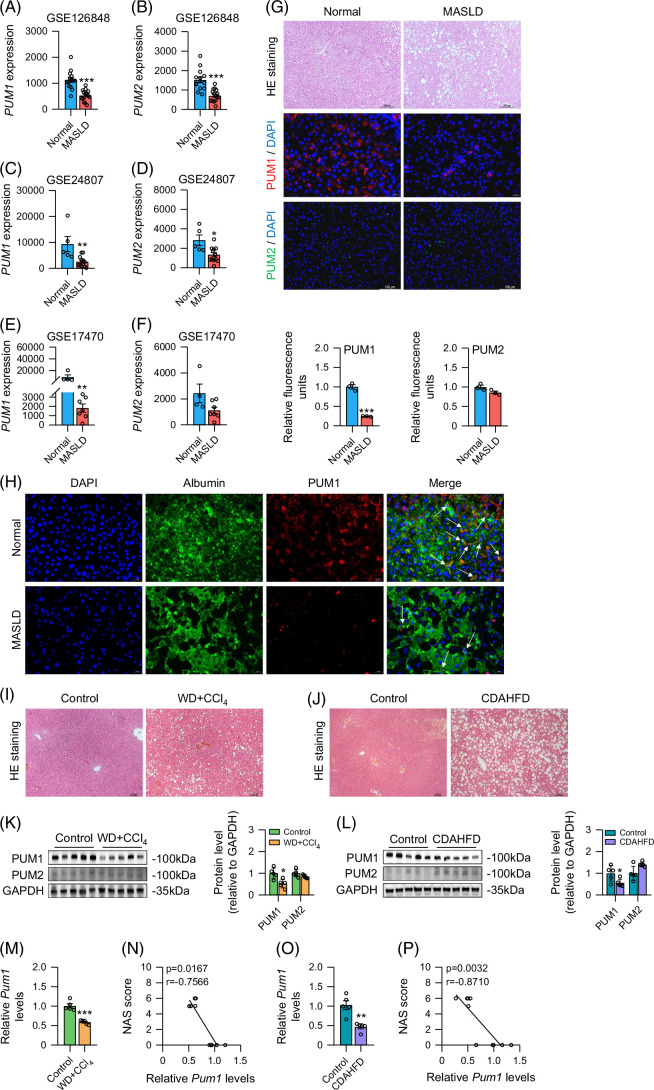
PUM1 was downregulated in MASLD. (A–F) *PUM1* and *PUM2* expression in the livers of MASLD patients and normal controls in the GSE126848 dataset (n=14 with normal control, n=16 with MASLD patients), GSE24807 dataset (n=5 with normal control, n=12 with MASLD patients), and GSE17470 dataset (n=4 with normal control, n=7 with MASLD patients). (G) Representing immunofluorescent images and fluorescence quantification of PUM1 and PUM2 expression in the liver tissues of MASLD patients and healthy controls (n=3). (H) Immunofluorescence staining showing Albumin (green) and PUM1 (red) colocalization in human liver tissues. (I, J) The representative images of HE staining of liver in mice. (K, L) Western blot analysis for PUM1 and PUM2 in the livers of mice (n=5). (M, O) Relative *Pum1* expression in the livers of MASLD mice (n=5). (N, P) Correlation analysis of relative *Pum1* expression with NAS score in MASLD mice. *, **, and *** denote *p* values <0.05, 0.01, and 0.001, respectively, in comparison to Normal groups or control groups. The white arrow indicates the colocalization of Albumin with PUM1. Abbreviations: CDAHFD, choline-deficient, l-amino acid–defined, high-fat diet; HE, hematoxylin and eosin; MASLD, metabolic dysfunction–associated steatotic liver disease; NAS, NAFLD activity score; WD, western diet.

To explore the role of PUM1 and PUM2 in the progression of MASLD, we analyzed their expression through 3 liver mRNA expression profiles of MASLD patients in the Gene Expression Omnibus (GEO) database. Analysis of patient data from 3 GEO datasets showed that PUM1 expression significantly decreased during MASLD (Figures 1A, C, E). In contrast, only 2 datasets (GSE126848 and GSE24807) revealed significant differences in PUM2 expression (Figures 1B, D, F). We further confirmed that patients with MASLD had lower hepatic PUM1 expression than healthy individuals, whereas PUM2 expression did not differ significantly (Figure 1G). Additionally, immunofluorescence experiments indicated that PUM1 and Albumin (a marker of hepatocytes) were colocalized (Figure 1H).

To bolster the GEO analysis, we measured PUM1/2 expression in 2 MASLD mouse models (Figures 1I–L). We then demonstrated that the livers of both WD+CCl_4_ and CDAHFD mice had significantly lower PUM1 protein expression levels than those of control mice, but PUM2 expression levels did not differ (Figures 1K, L). *Pum1* mRNA exhibited similar patterns to PUM1 (Figures 1M, O). In addition, Pum1 mRNA in the mouse liver was negatively correlated with the NAFLD activity score (NAS) (Figures 1N, P). To summarize, PUM1 downregulation during MASLD appears to promote disease progression.

### PUM1 knockdown aggravated liver injury in WD+CCl_4_ mice

**FIGURE 2:**
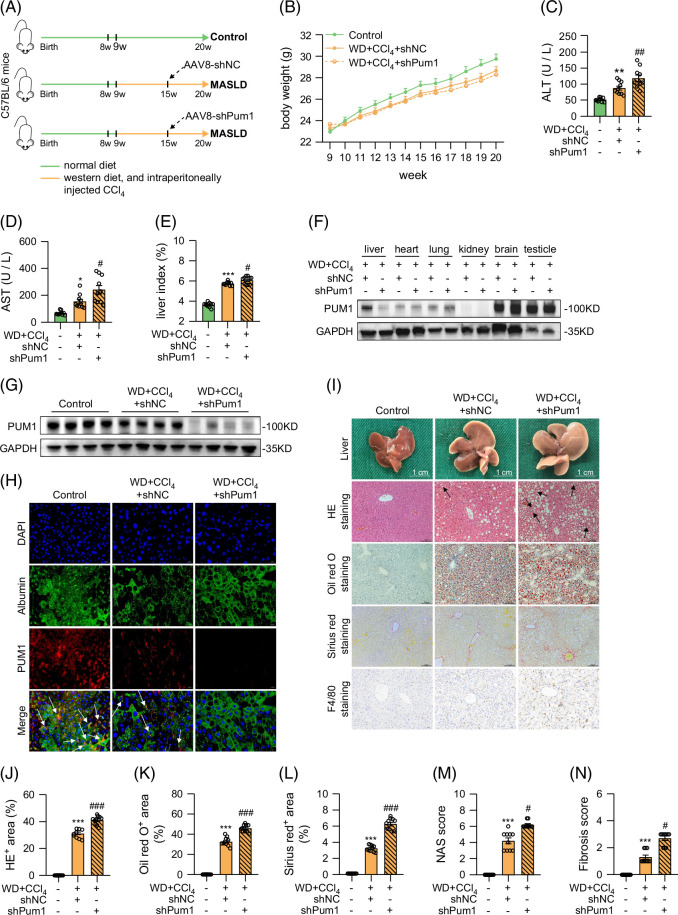
PUM1 knockdown aggravated liver injury in WD+CCl_4_ mice. (A) Schematic diagram for knockdown of PUM1 in WD+CCl_4_-induced MASLD model mice. (B) Body weight change curve of mice. (C–E) Serum ALT or AST concentrations and liver index in mice (n=10). (F) Western blot analysis for PUM1 expression in liver, heart, lung, kidney, brain, and testicle in MASLD mice. (G) Western blot analysis for PUM1 expression in mouse liver (n=4). (H) Immunofluorescence staining showing Albumin (green) and PUM1 (red) colocalization in mouse liver tissues. (I) The representative images of HE staining, Oil red O staining, Sirius red staining, and F4/80 staining of liver in mice. (J–N) HE^+^ area, Oil red O^+^ area, Sirius red^+^ area, NAS score, and liver fibrosis scores of mice in 3 groups (n=10). The white arrow indicates the colocalization of Albumin with PUM1. The black arrow indicates ballooning hepatocytes. *, **, and *** denote *p* values <0.05, 0.01, and 0.001, respectively, in comparison to control groups. #, ##, and ### denote *p* values <0.05, 0.01, and 0.001, respectively, in comparison to the WD+CCl_4_+shNC group. Liver index=liver weight/body weight × 100%. HE^+^ area (%)=area of fat vacuole/entire sample area × 100%. Oil red O^+^ area (%)=area of Oil red O staining/entire sample area × 100%. Sirius red ^+^ area (%)=area of Sirius red staining/entire sample area × 100%. Abbreviations: HE, hematoxylin and eosin; MASLD, metabolic dysfunction–associated steatotic liver disease; NAS, NAFLD activity score; WD, western diet.

We performed PUM1-knockdown experiments with injections of AAV8-shPum1 or AAV8-shNC into WD+CCl_4_ mice (Figure 2A) and monitored their body weight once a week (Figure 2B). After 11 weeks of WD+CCl_4_ intervention, serum ALT, AST, and liver weight index had increased significantly, a change that was enhanced with PUM1 knockdown (Figures 2C–E). PUM1 expression in the liver of MASLD model mice was significantly lower in the shPum1 group than in the shNC group, but no difference was observed in any other organ (heart, lung, kidney, brain, and testis) (Figures 2F, G). Immunofluorescence colocalization demonstrated that AAV8-shPum1 effectively reduced the expression of PUM1 in Albumin-labeled cells (Figure 2H). Additionally, the expression of PUM1 in αSMA-labeled cells was lower in the WD+CCl_4_+shPum1 group compared to the WD+CCl_4_+shNC group (Supplemental Figure S1A, http://links.lww.com/HC9/C39). Liver histology showed that PUM1 knockdown aggravated steatosis, ballooning, inflammation, and fibrosis in the liver of mice (Figures 2I–L). Besides, PUM1 knockdown increased NAS score (Figure 2M) and fibrosis score (Figure 2N). Together, our results show that knockdown of PUM1 aggravates liver injury in WD+CCl_4_-induced MASLD model mice.

### PUM1 knockdown aggravated liver injury in CDAHFD mice

**FIGURE 3:**
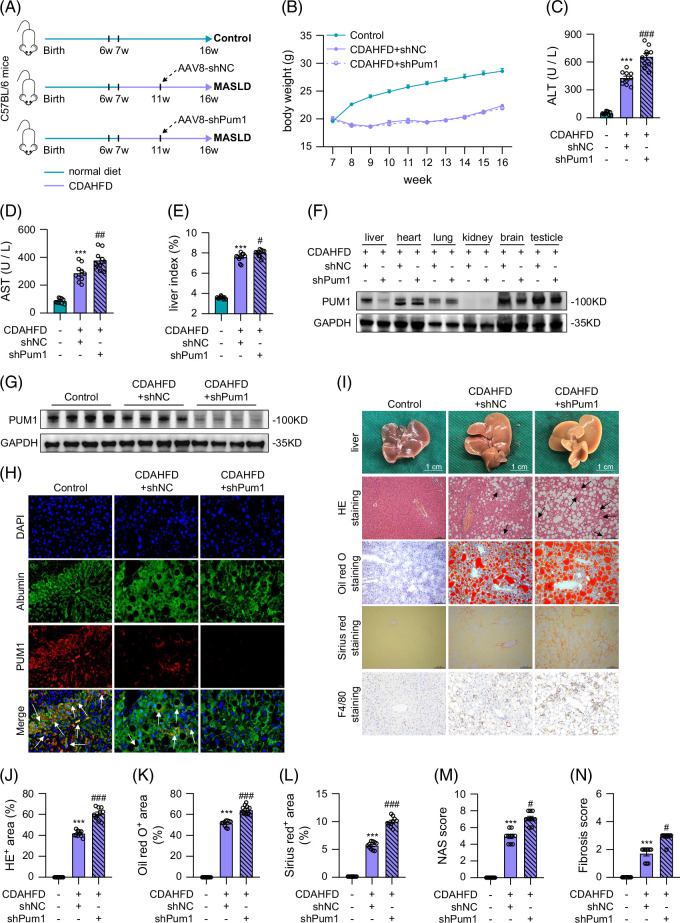
PUM1 knockdown aggravated liver injury in CDAHFD mice. (A) Schematic diagram for knockdown of PUM1 in CDAHFD-induced MASLD model mice. (B) Body weight change curve of mice. (C–E) Serum ALT or AST concentrations and liver index in mice (n=10). (F) Western blot analysis for PUM1 expression in liver, heart, lung, kidney, brain, and testicle in MASLD mice. (G) Western blot analysis for PUM1 expression in mouse liver (n=4). (H) Immunofluorescence staining showing Albumin (green) and PUM1 (red) colocalization in mouse liver tissues. (I) The representative images of HE staining, Oil red O staining, Sirius red staining, and F4/80 staining of liver in mice. (J–N) HE^+^ area, Oil red O^+^ area, Sirius red^+^ area, NAS score, and liver fibrosis scores of mice in 3 groups (n=10). The white arrow indicates the colocalization of Albumin with PUM1. The black arrow indicates ballooning hepatocytes. ***denotes *p* values <0.001 in comparison to control groups. #, ##, and ### denote *p* values <0.05, 0.01, and 0.001, respectively, in comparison to CDAHFD+shNC groups. Liver index=liver weight/body weight × 100%. HE^+^ area (%)=area of fat vacuole/entire sample area × 100%. Oil red O^+^ area (%)=area of Oil red O staining/entire sample area × 100%. Sirius red ^+^ area (%)=area of Sirius red staining/entire sample area × 100%. Abbreviations: CDAHFD, choline-deficient, l-amino acid–defined, high-fat diet; HE, hematoxylin and eosin; MASLD, metabolic dysfunction–associated steatotic liver disease; NAS, NAFLD activity score.

Next, we verified our findings in the CDAHFD mouse model with similar injections of AAV8-shPum1 or AAV8-shNC and measured the weight of the mice every week (Figures 3A, B). Nine weeks of CDAHFD dietary intervention caused a sharp rise in serum ALT, AST, and liver weight index, an increase that was heightened with PUM1 knockdown (Figures 3C–E). Additionally, PUM1 expression in the shPum1 group was downregulated only in the liver out of all measured organs (Figures 3F, G), indicating successful knockdown of PUM1 in the liver of the CDAHFD-induced MASLD model mice. Immunofluorescence colocalization demonstrated that AAV8-shPum1 effectively reduced the expression of PUM1 in Albumin-labeled cells (Figure 3H). Additionally, the expression of PUM1 in αSMA-labeled cells was lower in the CDAHFD+shPum1 group compared to the CDAHFD+shNC group (Supplemental Figure S1B, http://links.lww.com/HC9/C39). PUM1 knockdown aggravated steatosis, ballooning, inflammation, and fibrosis in the liver of mice (Figures 3I–L). PUM1 knockdown also resulted in increased NAS score (Figure 3M) and fibrosis score (Figure 3N). Taken together, we demonstrated that knockdown of PUM1 also aggravated liver injury in the CDAHFD-induced MASLD model mice.

### PUM1 regulated lipid deposition and lipotoxic death in steatotic hepatocytes

**FIGURE 4:**
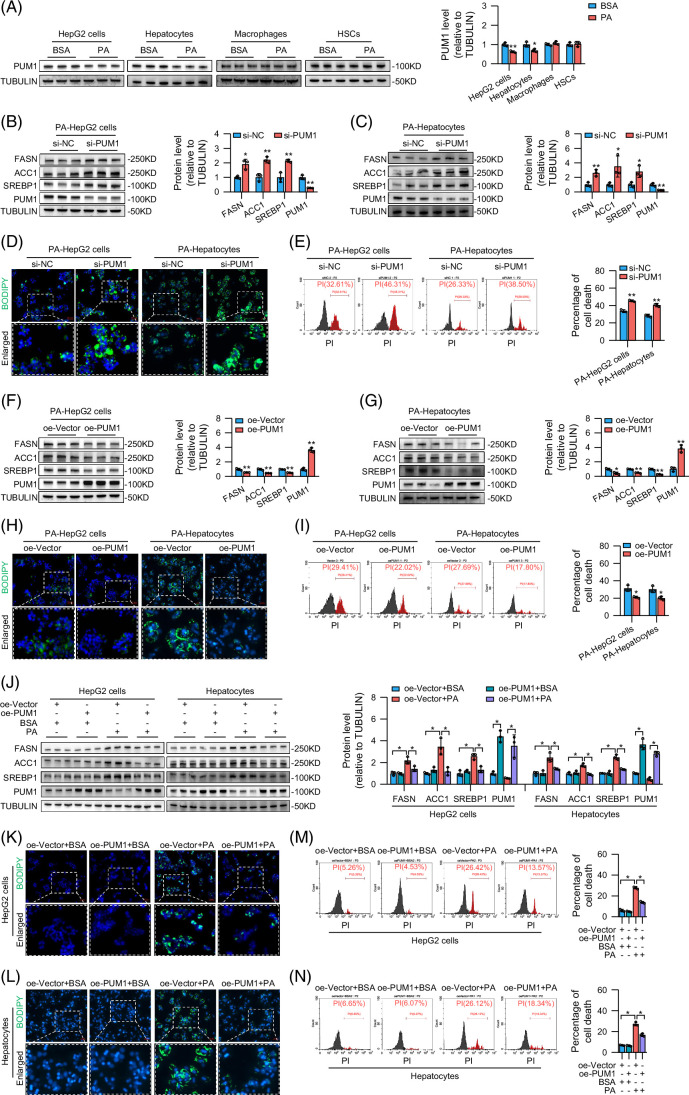
PUM1 regulated lipid deposition and lipotoxic death in steatotic hepatocytes. (A) Western blot analysis for PUM1 in PA-treated HepG2 cells, primary mouse hepatocytes, macrophages, and LX-2 cells (n=3). (B, C, F, G) Western blot analysis for PUM1, SREBP1, ACC1, and FASN expression in the steatosis hepatocytes treated with si-PUM1 or oe-PUM1 (n=3). (D, H) Representative images of BODIPY staining of steatotic hepatocytes treated with si-PUM1 or oe-PUM1. (E, I) PI staining of steatotic hepatocytes treated with si-PUM1 or oe-PUM1 (n=3). (J) Western blot analysis for PUM1, SREBP1, ACC1, and FASN expression in the HepG2 cells and primary mouse hepatocytes (n=3). (K, L) Representative images of BODIPY staining of HepG2 cells and primary mouse hepatocytes. (M, N) PI staining of HepG2 cells and primary mouse hepatocytes (n=3). *, **, and *** denote *p* values <0.05, 0.01, and 0.001, respectively, in comparison to the BSA, si-NC, and oe-Vector groups. Abbreviations: BSA, bovine serum albumin; FASN, fatty acid synthase; PA, palmitic acid; PI, propidium iodide.

We found that PUM1 expression was reduced in steatosis hepatocytes (PA-treated HepG2 cells and PA-treated mouse primary hepatocytes), but not in PA-treated macrophages and HSCs (Figure 4A). Western blot analysis demonstrated that knockdown of PUM1 resulted in increased expression of sterol regulatory-element binding protein 1 (SREBP1), acetyl-CoA carboxylase 1 (ACC1), and fatty acid synthase (FASN) in steatosis hepatocytes (Figures 4B, C). Knocking down PUM1 aggravated lipid deposition and lipotoxic death in steatosis hepatocytes (Figures 4D, E). Upregulation of PUM1 inhibited the expression of SREBP1, ACC1, and FASN (Figures 4F, G). Upregulation of PUM1 not only reduced lipid deposition but also reduced hepatocyte lipotoxic death (Figures 4H, I). PA promoted the expression of FASN, ACC1, and SREBP1 in HepG2 cells and primary mouse hepatocytes, while upregulation of PUM1 attenuated the promoting effect of PA on them (Figure 4J). Upregulation of PUM1 attenuated PA-induced lipid accumulation and lipotoxic death in HepG2 cells and primary mouse hepatocytes (Figures 4K–N).

### Hepatocyte-derived exosomes regulated the activation of HSCs via PUM1

**FIGURE 5:**
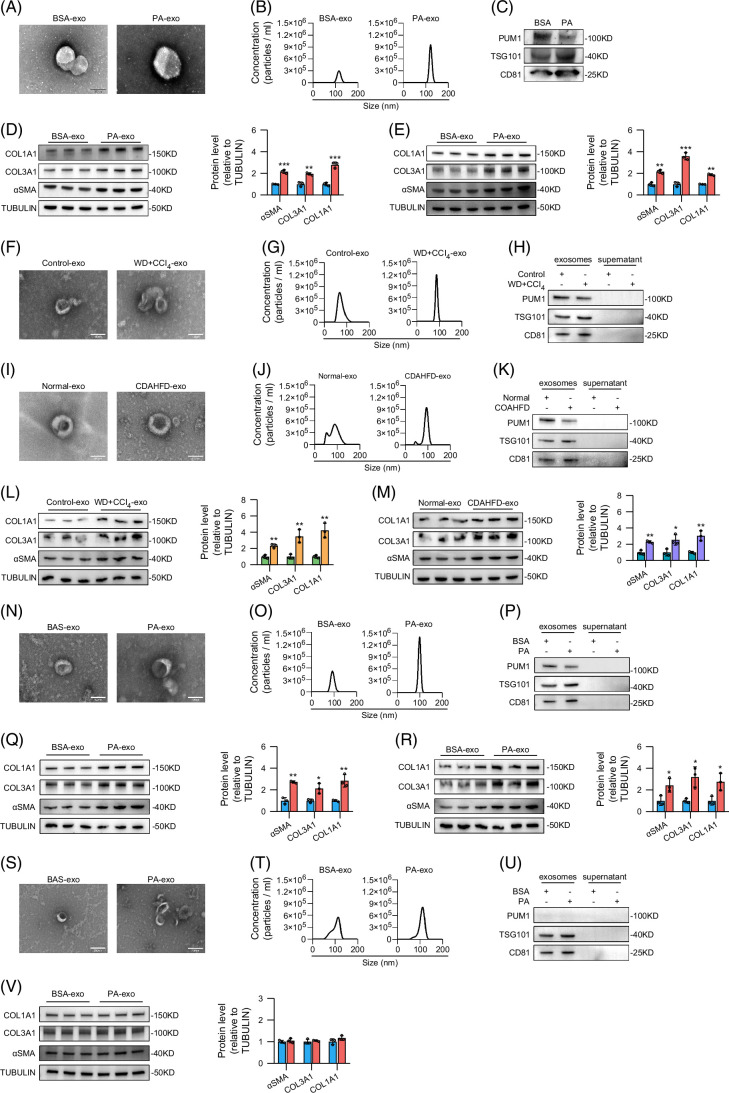
Hepatocyte-derived exosomes regulated the activation of HSCs via PUM1. (A) Transmission electron microscopy, (B) nanoparticle tracking analysis, and (C) western blot analysis were used to characterize HepG2 cell–derived exosomes. (D, E) Western blot analysis for αSMA, COL1A1, and COL3A1 expression in normal LX-2 cells or PUM1-knockdown LX-2 cells (n=3). (F, I) Transmission electron microscopy, (G, J) nanoparticle tracking analysis, and (H, K) western blot analysis were used to characterize exosomes from primary mouse hepatocytes. (L, M) Western blot analysis for αSMA, COL1A1, and COL3A1 expression in primary mouse HSCs (n=3). (N) Transmission electron microscopy, (O) nanoparticle tracking analysis, and (P) western blot analysis were used to characterize primary mouse hepatocyte-derived exosomes. (Q, R) Western blot analysis for αSMA, COL1A1, and COL3A1 expression in normal JS-1 cells or PUM1-knockdown JS-1 cells (n=3). (S) Transmission electron microscopy, (T) nanoparticle tracking analysis, and (U) western blot analysis were used to characterize exosomes from PUM1-knockdown primary mouse hepatocytes. (V) Western blot analysis for αSMA, COL1A1, and COL3A1 expression in normal JS-1 cells (n=3). *, **, and *** denote *p* values <0.05, 0.01, and 0.001, respectively, in comparison to BSA-exo, Control-exo, or Normal-exo. Abbreviations: BSA, bovine serum albumin; CDAHFD, choline-deficient, L-amino acid–defined, high-fat diet; Exo, exosomes; PA, palmitic acid; WD, western diet.

We isolated and characterized exosomes derived from steatotic hepatocytes (PA-treated HepG2 cells). Transmission electron microscopy showed that these exosomes had a typical saucer cup structure (Figure 5A). Nanoparticle tracking analysis showed that the average particle size of exosomes in the BSA (bovine serum albumin) group was 116.2 nm, while that in the PA group was 122.5 nm (Figure 5B). Western blot analysis showed that they expressed extracellular vesicle markers such as TSG101 and CD81 (Figure 5C). The expression of PUM1 in exosomes derived from steatotic hepatocytes was lower than that in the control group (Figure 5C). We further found that exosomes in the PA group promoted the expression of αSMA, COL1A1, and COL3A1 in both normal LX-2 cells (Figure 5D) and PUM1-knockdown LX-2 cells (Figure 5E). We further isolated and characterized exosomes from primary hepatocytes (Figures 5F–K) and serum (Supplemental Figures S2A–F, http://links.lww.com/HC9/C40) of MASLD mice induced by WD+CCl_4_ and CDAHFD, and characterized them (Figures 5F–K). The expression of PUM1 in exosomes was lower in both the WD+CCl_4_ group and the CDAHFD group compared to the control group (Figures 5H, K and Supplemental Figures S2C, F, http://links.lww.com/HC9/C40). Exosomes derived from primary hepatocytes of MASLD mice increased the expression of αSMA, COL1A1, and COL3A1 in HSCs (Figures 5L, M).

We further isolated exosomes derived from steatotic hepatocytes (PA-treated mouse primary hepatocytes) and characterized them (Figures 5N–P). Exosomes derived from PA-induced steatotic hepatocytes increased the expression of αSMA, COL1A1, and COL3A1 in both normal JS-1 cells (Figure 5Q) and PUM1-knockdown JS-1 cells (Figure 5R). We also isolated and characterized the exosomes from PUM1-knockdown primary mouse hepatocytes (Figure 5S–U). After knocking down PUM1, there was no significant difference in the effects of exosomes from the BSA group and the PA group on αSMA, COL1A1, and COL3A1 (Figure 5V).

### Knockdown of PUM1-activated HSCs and exacerbated liver fibrosis

**FIGURE 6:**
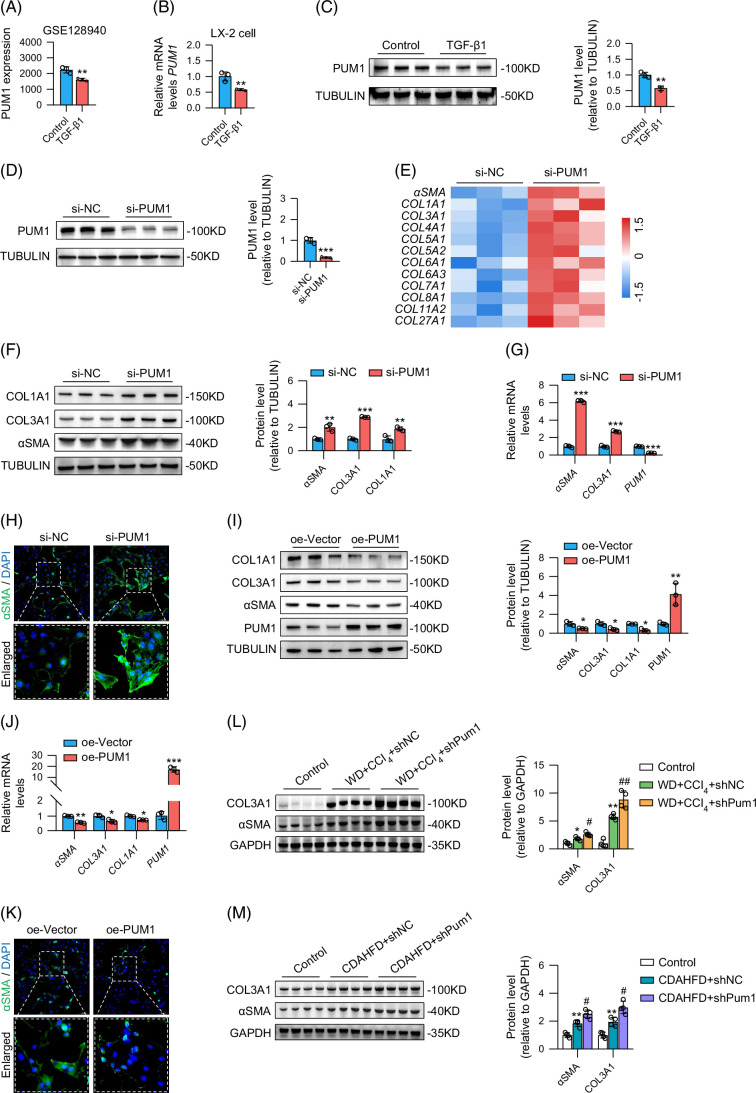
PUM1 regulates HSC activation. (A) The expression of *PUM1* in HSCs in the GSE128940 dataset (n=3). (B) The relative mRNA expression of *PUM1* in LX-2 cells in our in vitro experiments (n=3). (C, D) Western blot analysis for PUM1 expression in LX-2 cells (n=3). (E) Heatmap of RNA sequencing analysis for the expression of fibrosis-related genes in LX-2 cells. (F, I) Western blot analysis for COL1A1, COL3A1, and αSMA expression in LX-2 cells (n=3). (G, J) Relative mRNA levels of *PUM1*, *αSMA*, and *COL3A1* in LX-2 cells (n=3). (H, K) Representing immunofluorescent images of αSMA expression in LX-2 cells. (L, M) Western blot analysis for COL3A1 and αSMA expression in the liver of MASLD mice (n=4). ** and *** denote *p* values <0.01 and 0.001, respectively, in comparison to control, si-NC groups or oe-Vector groups. #, ##, and ### denote *p* values <0.05, 0.01, and 0.001, respectively, in comparison to the WD+CCl_4_+shNC group or CDAHFD+shNC groups. Abbreviations: CDAHFD, choline-deficient, l-amino acid–defined, high-fat diet; MASLD, metabolic dysfunction–associated steatotic liver disease; WD, western diet.

Analysis of the GEO dataset GSE128940 revealed that PUM1 expression was downregulated in HSCs activated by transforming growth factor-beta 1 (TGF-β1) (Figure 6A). We then confirmed this through RT-qPCR and western blot analysis, both PUM1 mRNA and protein levels were low in TGF-β1–activated HSCs (Figures 6B, C). We thus performed *PUM1* silencing experiments, transfecting si-PUM1 or negative controls (si-NC) into HSCs. Results from western blots indicated that PUM1 expression in the si-PUM1 group dropped by >70% (Figure 6D). Transcriptome sequencing of PUM1-knockdown HSCs revealed that numerous fibrosis-related genes (eg, *αSMA*, *COL1A1*, *COL3A1*) were more highly expressed in the si-PUM1 group than in the si-NC group (Figure 6E). In vitro experiments validated these findings, with PUM1 knockdown increasing αSMA, COL1A1, and COL3A1 protein expression in HSCs (Figure 6F). Moreover, *αSMA* and *COL3A1* mRNA levels were also upregulated in the si-PUM1 group than in the si-NC group (Figure 6G). Further confirming this effect, immunofluorescence experiments demonstrated that PUM1 knockdown resulted in enhanced αSMA fluorescent signals in HSCs (Figure 6H).

We also confirmed that upregulation of PUM1 inhibited the expression of αSMA, COL1A1, and COL3A1 (Figures 6I, J). Upregulation of PUM1 also resulted in weakening of the fluorescence signal of αSMA in HSCs (Figure 6K). Importantly, both intervention methods (WD+CCl_4_ or CDAHFD) promoted the expression of αSMA and COL3A1; the upregulation of αSMA and COL3A1 expression was further enhanced under PUM1 knockdown (Figures 6L, M). Together, we have demonstrated through various experiments that HSC activation is regulated by PUM1.

### PUM1 targets TPM4 mRNA in HSCs

**FIGURE 7:**
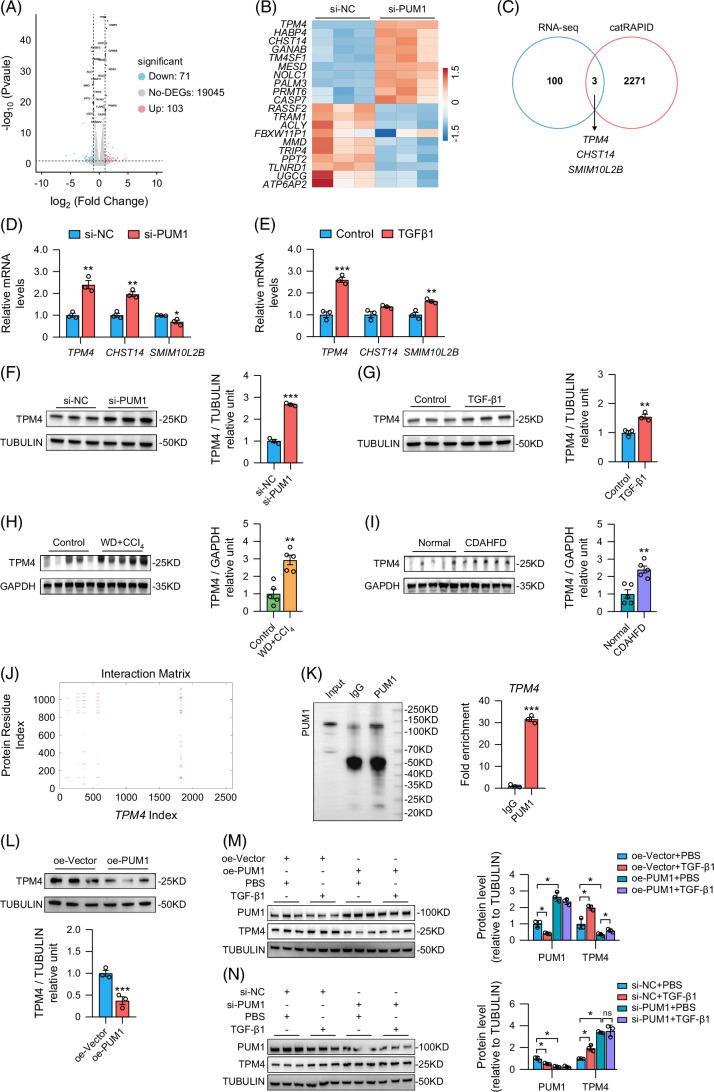
PUM1 targeted TPM4 mRNA in HSCs. (A) The volcano map of RNA sequencing analysis for differentially expressed genes in LX-2 cells. (B) The heatmap of RNA sequencing analysis for differentially expressed genes in LX-2 cells. (C) The intersection of the results of RNA sequencing analysis and the predicted results in the catRAPID database. (D, E) Relative mRNA expression of *TPM4*, *CHST14*, and *SMIM10L2B* in LX-2 cells (n=3). (F, G) Western blot analysis for TPM4 expression in LX-2 cells (n=3). (H, I) Western blot analysis for TPM4 in the livers of MASLD mice (n=5). (J) The binding sites of PUM1 protein and *TPM4* mRNA from the catRAPID database. (K) RNA immunoprecipitation (RIP) experiment for validation of the binding sites of PUM1 protein and *TPM4* mRNA (n=3). (L) Western blot analysis for TPM4 expression in LX-2 cells (n=3). (M, N) Western blot analysis for PUM1 and TPM4 expression in LX-2 cells (n=3). *, **, and *** denote *p* values <0.05, 0.01, and 0.001, respectively, in comparison to control groups, si-NC groups, or oe-Vector groups. Abbreviations: CDAHFD, choline-deficient, l-amino acid–defined, high-fat diet; MASLD, metabolic dysfunction–associated steatotic liver disease; WD, western diet.

After proving that knockdown of PUM1 can promote the activation of HSCs, we further explored the mechanism. We performed RNA sequencing to identify 103 upregulated and 71 downregulated genes in PUM1-knockdown versus no-knockdown HSCs (Figures 7A, B). Using the RNA-protein interaction database catRAPID, we predicted 2274 target mRNAs of PUM1 (Figure 7C). Of them, 3 target mRNAs (*TPM4*, *CHST14*, *SMIM10L2B*) were upregulated in the RNA-sequencing results of HSCs with PUM1 knocked down (Figure 7C). We then performed RT-qPCR on all 3 mRNAs and found that only *TPM4* mRNA expression increased in both PUM1-knockdown HSCs and TGF-β1–activated HSCs (Figures 7D, E). In addition, PUM1 knockdown increased TPM4 protein levels in HSCs (Figure 7F), and TPM4 concentrations also rose in TGF-β1–activated HSCs (Figure 7G). The expression of TPM4 in the liver of MASLD mice induced by WD+CCl_4_ or CDAHFD was higher than that in the control group (Figures 7H, I). In addition, compared to the control group, the expression of PUM1 in primary HSCs of mice in both the WD+CCl_4_ and CDAHFD groups was decreased, while the expression of TPM4 was increased (Supplemental Figures S3A, B, http://links.lww.com/HC9/C41). These outcomes suggest that *TPM4* is a target of PUM1.

The catRAPID analysis identified several binding sites between PUM1 and *TPM4* mRNA (Figure 7J). Through RIP experiments, we found that the PUM1 group exhibited *TPM4* enrichment ~30-fold higher than that in the IgG group (Figure 7K). Importantly, upregulation of PUM1 significantly inhibited the expression of TPM4 (Figure 7L). Our further experiments demonstrated that upregulation of PUM1 inhibited the expression of TPM4, while TGF-β1 attenuated the inhibitory effect of PUM1 on TPM4 (Figure 7M). After knocking down PUM1, the expression of TPM4 was not affected by TGF-β1 (Figure 7N). Overall, PUM1 can directly bind to *TPM4* mRNA, thereby regulating the translation of TPM4 in HSCs.

### PUM1 targeted TPM4 to regulate HSCs activation

**FIGURE 8:**
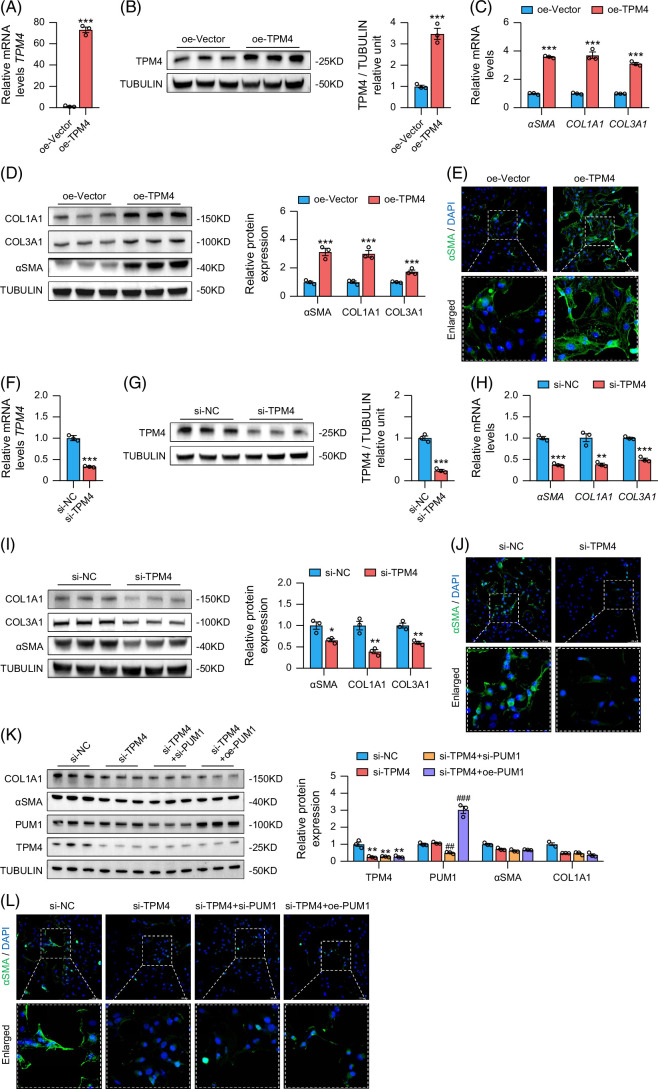
PUM1 targeted TPM4 to regulate HSCs activation. (A, B) RT-qPCR analysis and western blot analysis for the overexpression effect of TPM4 in LX-2 cells (n=3). (C, D) RT-qPCR analysis and Western blot analysis for αSMA, COL1A1, and COL3A1 in LX-2 cells (n=3). (E) Representing immunofluorescence images for αSMA in LX-2 cells. (F, G) RT-qPCR analysis and Western blot analysis for TPM4 expression in LX-2 cells (n=3). (H, I) RT-qPCR analysis and Western blot analysis for αSMA, COL1A1, and COL3A1 in LX-2 cells (n=3). (J, L) Representing immunofluorescence images for αSMA in LX-2 cells. (K) Western blot analysis for COL1A1, αSMA, PUM1, and TPM4 in LX-2 cells (n=3). *, **, and *** denote *p* values <0.05, 0.01, and 0.001, respectively, in comparison to si-NC or oe-Vector groups. ### denote *p* values <0.001, respectively, in comparison to si-TPM4 groups. Abbreviation: RT-qPCR, quantitative reverse transcription polymerase chain reaction.

To investigate the role of TPM4 in HSCs activation, we transfected a plasmid overexpressing TPM4 (oe-TPM4) into HSCs and observed an 80-fold increase in *TPM4* mRNA levels among the oe-TPM4 cells (Figure 8A). The corresponding TPM4 protein concentration also increased significantly in oe-TPM4 HSCs (Figure 8B). We also observed upregulation of αSMA, COL1A1, and COL3A1 mRNA and protein in the oe-TPM4 group (Figures 8C, D). Immunofluorescence staining further validated αSMA upregulation in TPM4-overexpressing HSCs (Figure 8E). Together, these data indicate that TPM4 overexpression promotes HSC activation.

After transfecting si-TPM4 into HSCs, RT-qPCR and western blotting confirmed the success of TPM4 knockdown in the si-TPM4 group (Figures 8F, G). Knocking down TPM4 caused a significant downregulation of αSMA, COL1A1, and COL3A1 in HSCs (Figures 8H, I). Immunofluorescence staining further confirmed that αSMA expression is reduced in TPM4-knockdown HSCs (Figure 8J). Thus, TPM4 knockdown inhibits HSC activation.

Upregulation or knockdown of PUM1 in TPM4-knockdown HSCs did not alter αSMA and COL1A1 protein concentrations (Figure 8K), nor did it change αSMA fluorescence intensity (Figure 8L). As previously described, knocking down PUM1 promoted HSC activation (Figures 6F–H), while upregulating PUM1 inhibited the activation of HSCs (Figures 6I–K). This promotion or inhibition was abolished under TPM4 knockdown. Taken together, PUM1 acts on TPM4 to regulate HSC activation.

## DISCUSSION

At present, effective drugs for the treatment of MASLD are still very limited, largely due to the incomplete understanding of its pathogenesis. This study successfully confirmed that PUM1 expression is downregulated in MASLD, using patient liver samples and 2 animal models. We verified the involvement of PUM1 with knockdown experiments in our mouse models that aggravated liver damage from MASLD. In addition to these in vivo data, our in vitro experiments in steatotic hepatocytes demonstrated that knockdown of PUM1 aggravated lipid deposition, while upregulation of PUM1 improved lipid deposition. Exosomes derived from steatotic hepatocytes promoted activation of HSCs. Finally, we elucidated a major mechanism underlying activation of HSCs; specifically, knockdown of PUM1 resulted in increased translation of TPM4, and overexpression of TPM4 induced activation of HSCs.

PUM1 is a conserved RNA-binding protein that acts on target RNA through the PUF domain.^8^ Abnormal expression or dysfunction of PUM1 is associated with various disorders, including excess inflammation,^14^^,^^28^ malignant tumors (such as gastric cancer, colorectal cancer, and pancreatic cancer),^9^^,^^10^^,^^17^^,^^18^ and neurological diseases.^29^ PUM1 has also been implicated in the cell cycle and tissue development.^12^^,^^30^ Here, we provided evidence of a novel role for PUM1—its involvement in MASLD. We not only confirmed that the expression of PUM1 is downregulated during the progression of MASLD, but also found that the expression of PUM1 in steatotic hepatocytes was reduced. With the downregulation of PUM1, the expression of SREBP1, ACC1, and FASN in hepatocytes increased. Upregulation of PUM1 inhibited the expression of these fatty acid synthesis-related proteins. The reduction of PUM1 led to lipid deposition and aggravated liver injury, while upregulation of PUM1 improved lipid deposition and hepatocyte lipotoxic death. Therefore, PUM1 is involved in the progression of MASLD by regulating lipid metabolism pathways.

Sustained activation of HSCs is closely linked to liver injury in MASLD. Studies have shown that hepatocyte-derived exosomes promote the activation of HSCs.^31^ We found that exosomes derived from steatotic hepatocytes promoted the activation of HSCs, and the mechanism was that the content of PUM1 in exosomes derived from steatotic hepatocytes decreased. We further upregulated PUM1 and found that the activation of HSCs was inhibited by the increase of PUM1.

Tropomyosin (TPM) has been implicated in the activation of HSCs.^32^ TPM4 is 1 of the 4 protein isoforms (TPM1, TPM2, TPM3, and TPM4) of TPM in mammals.^33^ TPM4, as an actin-binding protein, binds to actin and participates in regulating cytoskeletal changes.^34^ Additionally, TPM4 condensates recruit glycolytic enzymes such as HK2, PFKM, and PKM2, thereby regulating the glycolysis process.^35^ Hepatic TPM4 expression was higher in a mouse model of CCl_4_-induced liver fibrosis than in control mice.^36^ TPM4 is also involved in regulating epithelial–mesenchymal transition, a key mechanism behind liver fibrosis development.^37^^,^^38^ Our study further confirmed the exact role of TPM4 in HSCs activation, its overexpression directly activates HSCs, whereas its knockdown inhibits HSCs activation. We also demonstrated that *TPM4* is a direct target of PUM1. In normal HSCs, PUM1 inhibits TPM4 translation, and when PUM1 is downregulated, the resultant upregulation of TPM4 leads to HSC activation. Therefore, TPM4 may serve as a potential therapeutic target for MASLD.

In conclusion, PUM1 downregulation promotes MASLD progression, whereas upregulation of PUM1 improves MASLD, reflecting its critical role in the disease. Mechanistically, PUM1 reduced lipid deposition and lipotoxic death in hepatocytes and hepatocyte-derived exosomes carrying PUM1 inhibited the activation of HSCs. PUM1 directly binds to *TPM4* mRNA, thereby inhibiting TPM4 translation to reduce HSCs activation. Our findings uncovered a novel role of PUM1 in MASLD, improved the current understanding of MASLD progression, and provided potential clinical targets for advancing treatment strategies.

## Supplementary Material
