# Supplementary material for: Hepatocyte-derived Pumilio1-enriched exosomes inhibit HSC activation by suppressing tropomyosin-4 translation
Source: Hepatol Commun. 2025 Jul 14;9(8):e0759. doi: 10.1097/HC9.0000000000000759 (PMC12263002; doi:10.1097/HC9.0000000000000759)
Supplement: Supplementary file 1 [file hc9-9-e0759-s001.docx]

**Supplementary materials and methods**

**Hepatocyte-derived Pumilio1-enriched exosomes inhibit hepatic stellate cell activation by suppressing tropomyosin-4 translation**

Zhiping Wan ^1, 2, 4^, Xiang Cai ^1, 2, 4^, Xiaoquan Liu ^1, 2, 4^, Haoqi Chen ^2, 3, 4^, Xiaoman Chen ^1, 2^, Xiaoan Yang ^1, 2,^ Qingqing Feng ^1, 2^, Hong Cao ^1, *^, Qiyi Zhao ^1, 2, *^, Hong Deng ^1, 2, *^

^1^ Department of Infectious Diseases, the Third Affiliated Hospital of Sun Yat-sen University, Guangzhou 510630, China.

^2^ Guangdong Key laboratory of Liver Disease Research, the Third Affiliated Hospital of Sun Yat-sen University, Guangzhou 510630, China.

^3^ Department of Hepatic Surgery, Liver Transplantation, the Third Affiliated Hospital of Sun Yat-sen University, Guangzhou 510630, China.

^4^ Co-first author: Zhiping Wan, Xiang Cai, Xiaoquan Liu and Haoqi Chen

***Corresponding authors:**

Hong Deng, M.D., Department of Infectious Diseases, Guangdong Key laboratory of Liver Disease Research, the Third Affiliated Hospital of Sun Yat-sen University, Guangzhou, China. E-mail: dhong@mail.sysu.edu.cn.

Qiyi Zhao, Ph.D., Department of Infectious Diseases, Guangdong Key laboratory of Liver Disease Research, the Third Affiliated Hospital of Sun Yat-sen University, Guangzhou, China. E-mail: zhaoqyi@mail.sysu.edu.cn.

Hong Cao, M.D., Department of Infectious Diseases, the Third Affiliated Hospital of Sun Yat-sen University, Guangzhou, China. E-mail: caohong@mail.sysu.edu.cn.

***Immunofluorescence staining***

Liver tissues were embedded in paraffin, deparaffinized, and rehydrated. Tissue sections were then incubated overnight with primary antibodies for PUM1 (1:100, #ab92545, Abcam, Cambridge, MA, USA) or PUM2 (1:100, #ab92390, Abcam) at 4 ºC. Next, they were incubated with fluorophore-conjugated secondary antibody for 1 hour at 37 ºC, followed by DAPI solution for 5 minutes. Images were captured under a fluorescence microscope (Leica, Wetzlar, Germany). For antibody details, see Supplementary Table S1.

For PUM1/Albumin co-staining or PUM1/αSMA co-staining, sections were first incubated overnight with anti-PUM1 antibody (1:100, #ab92545, Abcam) at 4 ºC, then incubated at room temperature with the corresponding fluorophore-conjugated secondary antibody for 1 h. Tissue sections were microwaved for antigen retrieval, incubated overnight with anti-Albumin antibody (1:500, #ab207327, Abcam) or anti-αSMA antibody (1:1500, #ab32575, Abcam) at 4 ºC, and then for 1 h with the corresponding fluorophore-conjugated secondary antibody incubation at room temperature. Sections were washed three times before DAPI addition and incubation for 10 min at room temperature. Images were captured under a fluorescence microscope (Leica, Wetzlar, Germany). For antibody details, see Supplementary Table S1.

***Immunohistochemistry staining***

After incubating the liver tissue sections with the anti-F4/80 antibody (1:2000, #ab300421, Abcam) overnight (4°C), they were incubated with the HRP-conjugated secondary antibody (1:200, #GB23303, Servicebio, room temperature, 50 minutes). Finally, staining was performed using DAB (#G1211, Servicebio) staining solution, followed by counterstaining with hematoxylin. Images were captured under a fluorescence microscope (Leica, Wetzlar, Germany). For antibody details, see Supplementary Table S1.

***Alanine transaminase (ALT) and aspartate transaminase (AST) measurement***

Levels of ALT and AST in the serum of C57BL/6 mice were determined using ALT assay kits (#C009; Nanjing Jiancheng, Nanjing, China) and AST assay kits (#C010, Nanjing Jiancheng), respectively. All assays were performed according to the manufacturers’ instructions.

***Cell BODIPY staining analysis***

The cells were treated with 4% paraformaldehyde for 15 minutes to fix the cells. The cells were then treated with 0.1% Triton-X100 (#T9284, Sigma-Aldrich, Saint Louis, MO, USA) for 30 min and stained with 5 μM BODIPY reagent (#HY-W090090; Med Chem Express, Monmouth Junction, NJ, USA) for 30 min. Finally, all images were collected using a microscope.

***Western blot analysis***

Proteins from tissues and cells were extracted using radioimmunoprecipitation assay (RIPA) buffer (#89900; Thermo Fisher Scientific, Waltham, MA, USA) containing a protease inhibitor and phosphatase inhibitor cocktail (#78440, Thermo Fisher Scientific). Protein samples were then mixed with a loading buffer (#G4552; GBCBIO, Guangzhou, China) and placed in boiling water for 10 minutes. The protein samples were separated using sodium dodecyl sulphate-polyacrylamide gel electrophoresis (SDS-PAGE) and transferred to polyvinylidene fluoride (PVDF) membranes that were blocked with 5% skim milk and then incubated with primary antibodies overnight at 4 °C. The membranes were then incubated with secondary antibodies at room temperature for 60 minutes, and the protein bands were detected using a chemiluminescence instrument (Tanon, Shanghai, China). The bands were analyzed using ImageJ software. All antibody information is provided in Table S1.

***Quantitative reverse transcription polymerase chain reaction (RT-qPCR) analysis***

RNA was extracted from rat and mouse liver tissues and cells using TRIzol reagent (#15596018; Invitrogen, Carlsbad, CA, USA). The RNA was then used as a template to synthesize complementary DNA using Reverse Transcriptase Reagent (#RR036; Takara, Kyoto, Japan), and TB Green Premix Ex Taq II (#RR820A, Takara) was used for fluorescence quantification of the RT-qPCR product. For quantification, β-actin served as an internal reference for mRNA. All primer sequences are listed in Table S2.

***Cell immunofluorescence analysis***

The cells were fixed with 4 % paraformaldehyde, permeabilized with 0.5 % Triton-X-100 (#T9284, Sigma-Aldrich, St Louis, Missouri, USA), and blocked with 5 % BSA (#G1211 Servicebio) for 30 min. The cells were then incubated with the primary antibody for αSMA (1:1500, #ab32575, Abcam) overnight at 4 ºC. Next, they were incubated with fluorophore-conjugated secondary antibody for 1 h, followed by DAPI solution for 5 min at 37ºC. Images were obtained using a Leica fluorescence microscope (Leica, Wetzlar, Germany).

| **Table S1. The antibody information used in this study.** | | | | |
| --- | --- | --- | --- | --- |
| **Antibodies** | **Dilution** | **Experiment** | **Manufacturer** | **Catalog number** |
| PUM1 | 1:1000 | Western blot | Abcam | ab92545 |
| PUM2 | 1:1000 | Western blot | Abcam | ab92390 |
| GAPDH | 1:2000 | Western blot | Cell Signaling Technology | #2118 |
| SREBP1 | 1:1000 | Western blot | Santa Cruz | sc-525476 |
| FASN | 1:1000 | Western blot | Abcam | ab128856 |
| ACC1 | 1:1000 | Western blot | Abcam | ab45174 |
| TSG101 | 1:500 | Western blot | Signalway Antibody | #49270 |
| CYP2E1 | 1:1500 | Western blot | Abcam | # ab28146 |
| CD81 | 1:500 | Western blot | Abcam | ab109201 |
| COL3A1 | 1:1000 | Western blot | Abcam | ab184993 |
| αSMA | 1:1000 | Western blot | Abcam | ab32575 |
| COL1A1 | 1:1000 | Western blot | Abcam | ab138492 |
| β-ACTIN | 1:1000 | Western blot | Cell Signaling Technology | #4967 |
| TUBULIN | 1:2000 | Western blot | Cell Signaling Technology | #2128 |
| TPM4 | 1:1000 | Western blot | Abcam | ab181085 |
| PUM1 | 1:20 | Immunoprecipitation | Thermo Fisher Scientific | #MA5-41264 |
| PUM1 | 1:100 | Immunofluorescence | Abcam | ab92545 |
| PUM2 | 1:100 | Immunofluorescence | Abcam | ab92390 |
| Albumin | 1:500 | Immunofluorescence | Abcam | ab207327 |
| αSMA | 1:1500 | Immunofluorescence | Abcam | ab32575 |

| **Table S2. Sequences of primers used for RT-qPCR assays.** | | |
| --- | --- | --- |
| **Species** | **Gene** | **Sequences of primers** |
| *Mouse* | *Pum1* | Forward: ATCAGATCATTCAGTTTCCCACC  Reverse: TAACGCCTAGTCCCCCACTC |
| *Mouse* | *β-Actin* | Forward: TGTGGATCGGTGGCTCCATCCT  Reverse: AAACGCAGCTCAGTAACAGTCCGC |
| *Human* | *PUM1* | Forward: ATGAGCGTTGCATGTGTCTTG  Reverse: GTAGTCCACCATAGCGTCGTC |
| *Human* | *COL1A1* | Forward: CACCAATCACCTGCGTACAG  Reverse: GCAGTTCTTGGTCTCGTCAC |
| *Human* | *COL3A1* | Forward: TTGAAGGAGGATGTTCCCATCT  Reverse: ACAGACACATATTTGGCATGGTT |
| *Human* | *αSMA* | Forward: CTATGAGGGCTATGCCTTGCC  Reverse: GCTCAGCAGTAGTAACGAAGGA |
| *Human* | *TPM4* | Forward: TCCGACGCTGAAGGTGATGTG  Reverse: CCCTGTCCAACTCCTCCTCAAC |
| *Human* | *CHST14* | Forward: TGACCGTTACCGCTTCCTCTAC  Reverse: CTTCATCACCCGCTTCCAGTTAG |
| *Human* | *SMIM10L2B* | Forward: ACGCTGCTCACCTTCTTCGAC  Reverse: ATCACCGAGGCCACGATGTAG |
| *Human* | *β-ACTIN* | Forward: TGTGGATCGGTGGCTCCATCCT  Reverse: AAACGCAGCTCAGTAACAGTCCGC |
