# Supplementary figures and images for: Hepatocyte-derived Pumilio1-enriched exosomes inhibit HSC activation by suppressing tropomyosin-4 translation
Source: Hepatol Commun. 2025 Jul 14;9(8):e0759. doi: 10.1097/HC9.0000000000000759 (PMC12263002; doi:10.1097/HC9.0000000000000759)

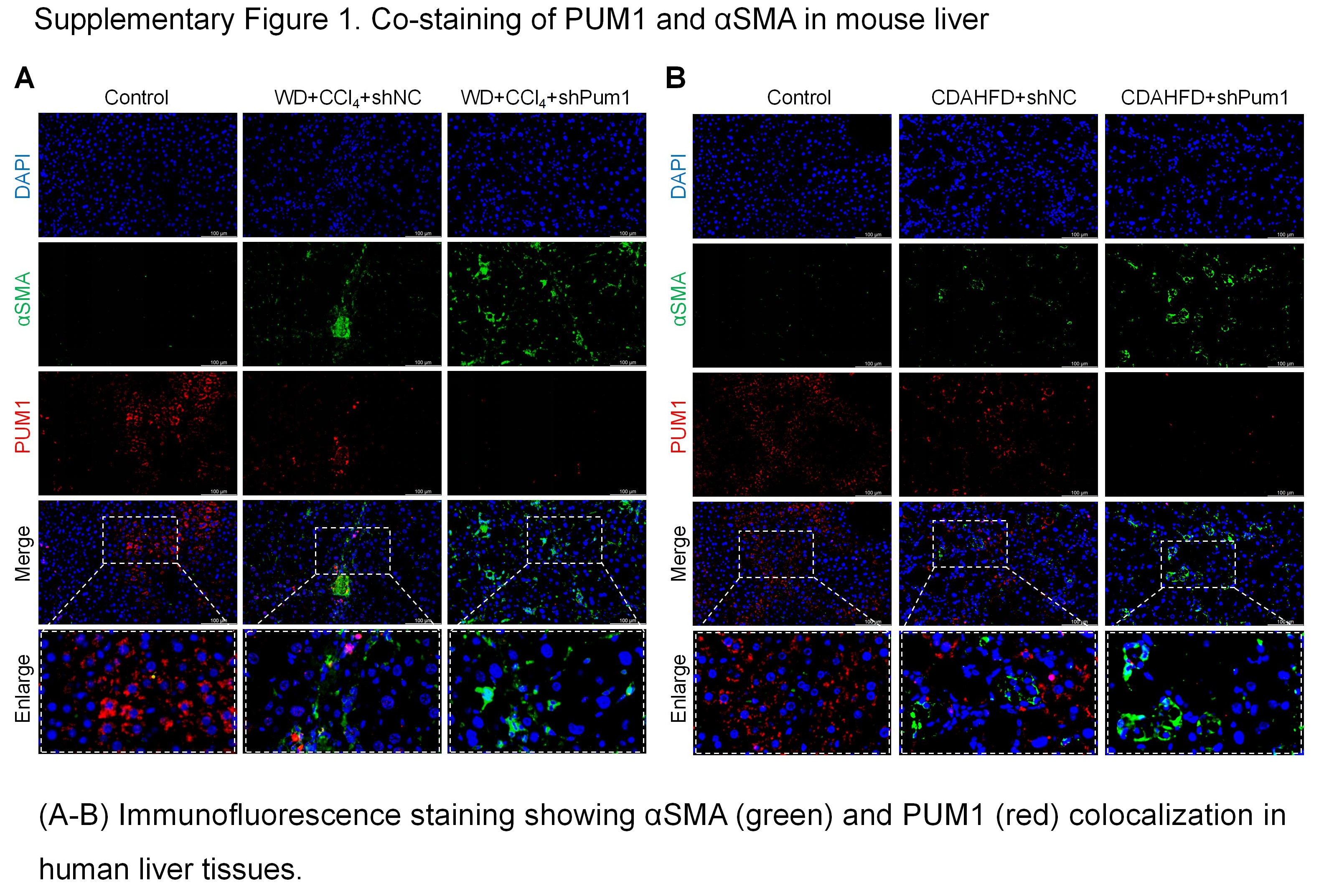

Supplement: Supplementary file 2 [file hc9-9-e0759-s002.jpg]

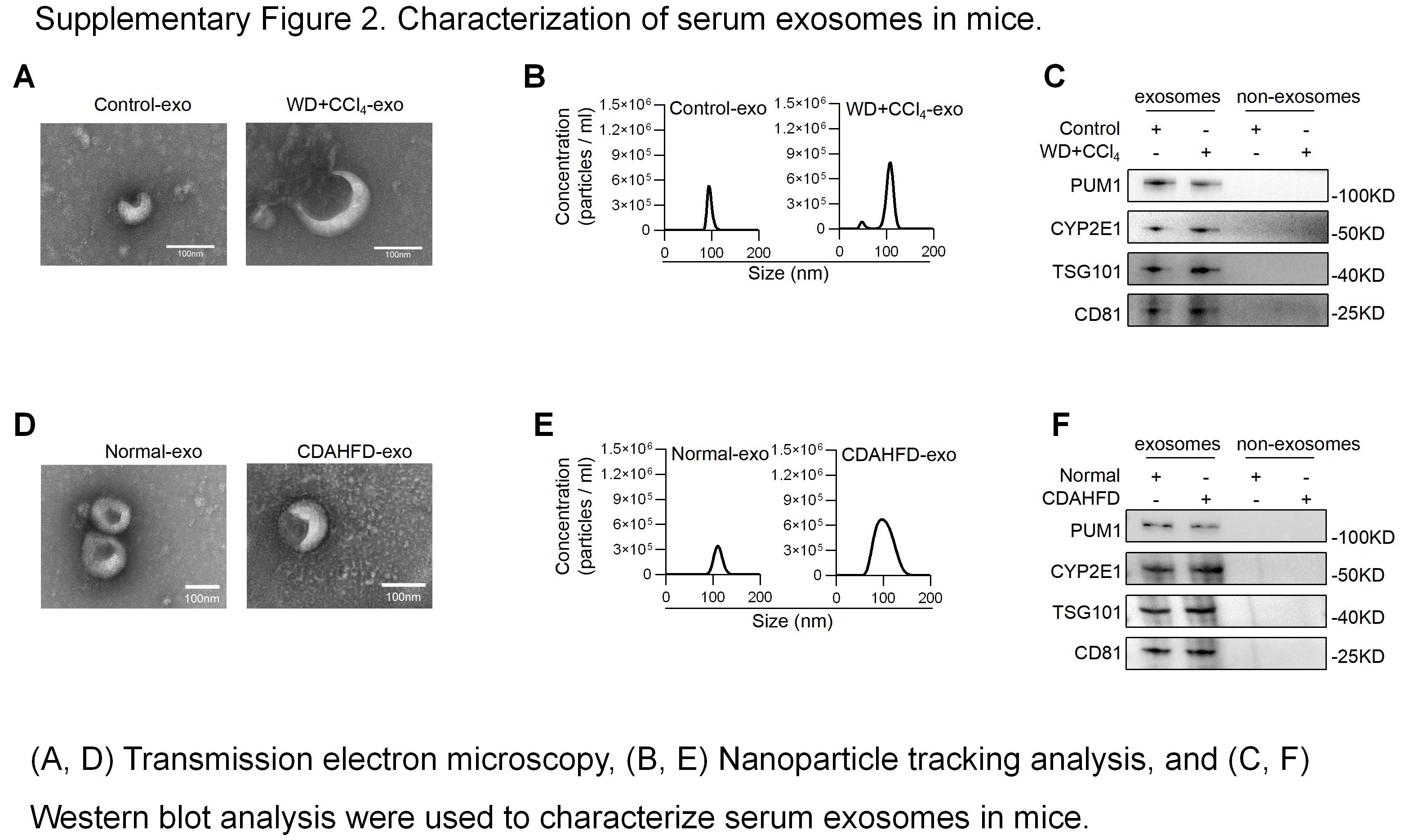

Supplement: Supplementary file 3 [file hc9-9-e0759-s003.jpg]

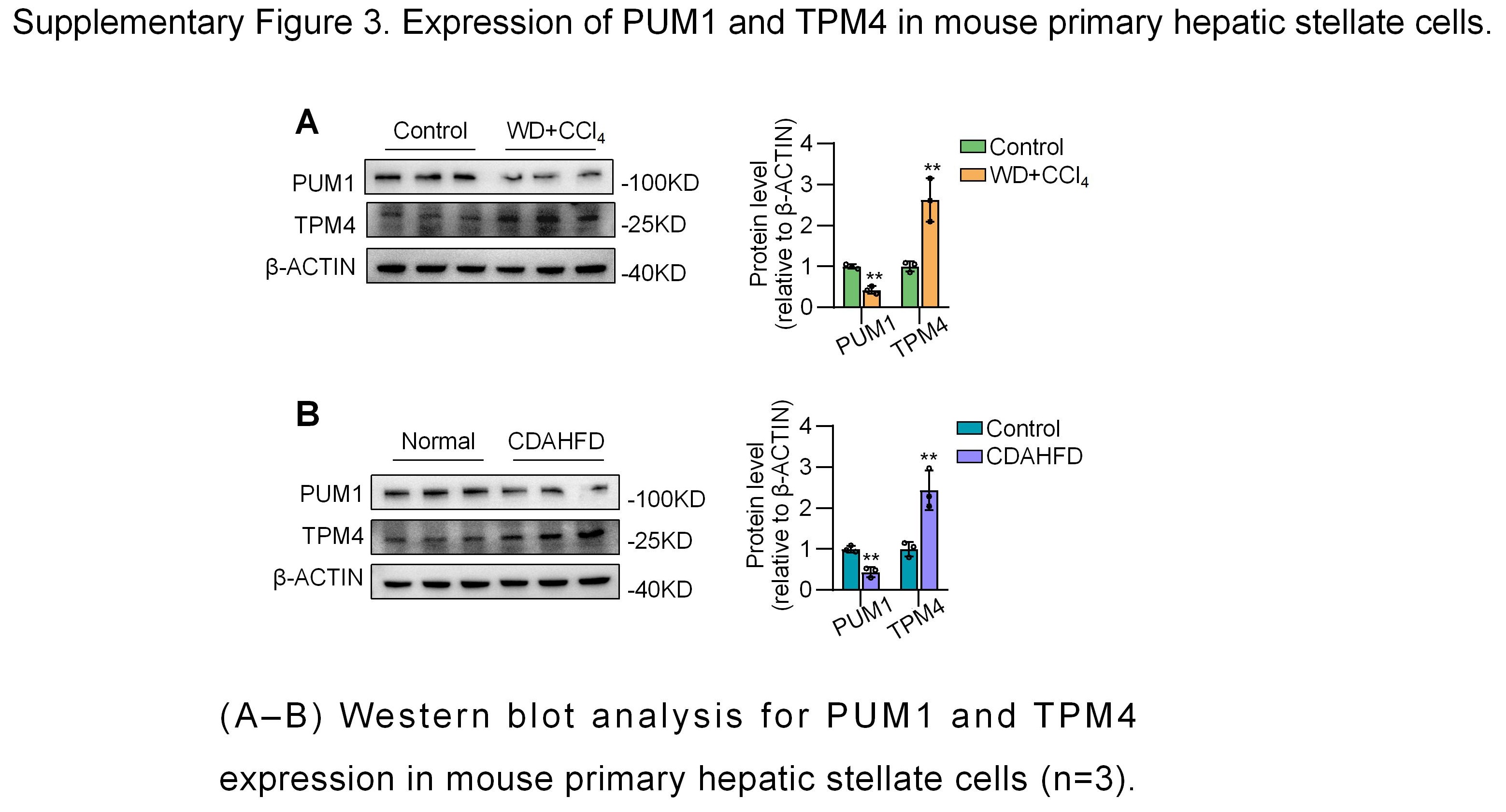

Supplement: Supplementary file 4 [file hc9-9-e0759-s004.jpg]
